# An Unusual Case of Streptococcus anginosus Endocarditis in a Healthy Host With Bicuspid Aortic Valve

**DOI:** 10.7759/cureus.13171

**Published:** 2021-02-06

**Authors:** Kai-Ming Chang, Sharon L Hsieh, Robin Koshy

**Affiliations:** 1 Division of Infectious Diseases, Department of Medicine, Donald and Barbara Zucker School of Medicine at Hofstra/Northwell Health, Manhasset, USA; 2 Internal Medicine, University of Miami Leonard M. Miller School of Medicine, Miami, FL, USA

**Keywords:** s. anginosus, streptococcus anginosus group, viridans streptococci, endocarditis, streptococcal milleri group, s. anginosus endocarditis, infective endocarditis, streptococcal bacteremia, aortic valve insufficiency, bicuspid aortic valve

## Abstract

*Streptococcus anginosus *group (SAG) is a subgroup of viridans streptococci and can be found ubiquitously in normal human flora. SAG is known to form invasive pyogenic infection when it becomes pathogenic. Yet, SAG is a very rare cause of endocarditis, and there is a dearth of case reports on this topic. We present a rare case of native bicuspid aortic valve endocarditis secondary to *S. anginosus* that caused aortic insufficiency and ascending aortic aneurysm. To our knowledge, this is the first well-documented case report of community-acquired *S. anginosus* endocarditis on a bicuspid aortic valve in an immunocompetent patient. The patient first presented with cough that was likely due to bronchus irritation from a 5.5 x 5.2 cm ascending aortic aneurysm. He underwent aortic valve replacement with bovine bioprosthesis and ascending aortic aneurysm repairment and was treated with a two-week regimen of IV ceftriaxone and gentamicin followed by another four weeks of IV ceftriaxone. He was eventually discharged to a rehabilitation facility. SAG is usually susceptible to beta-lactam antibiotics. The prognosis of SAG infection is usually good, but progression to bacteremia carries a poor outcome.

## Introduction

Streptococcus *anginosus* group (SAG), formally known as *Streptococcal milleri* group, consists of *S. anginosus*, *S. constellatus*, and *S. intermedius*. It was first isolated by Guthof in 1956 from dental abscesses [1]. They are frequently found in different parts of the human tissues and are considered harmless normal flora. When the protective skin barriers and tissues are disrupted, they can be opportunistic pathogens and are known to form invasive abscesses. *Streptococcus anginosus* is a very rare causative organism for bacteremia and infective endocarditis (IE). Bicuspid aortic valve is a risk factor for IE. However, *S. anginosus* endocarditis on a bicuspid aortic valve is relatively uncommon [2]. Here, we present such a case and review the treatment and prognosis of patients with *S. anginosus* endocarditis.

## Case presentation

This is a case of a 53-year-old male who had been healthy until the onset of dry cough, fever, and weight loss that started four months prior to admission to a suburban hospital in New York. He had no hypertension, diabetes, or recurrent infection. He emigrated from Columbia to the United States about a year ago. He worked in a restaurant and lived in an apartment with roommates with no pets at home. He had a remote smoking history of more than 30 years ago and had no known allergy. Apart from growing up in an endemic region, he had no other known exposure to tuberculosis. During his outpatient visit, he had been worked up extensively, which include blood tests, urinalysis, chest X-ray (CXR), and echocardiogram. He had tried a course of antibiotics for presumed pneumonia, a course of antihistamine agent, and corticosteroids without showing much improvement.

**Figure 1:**
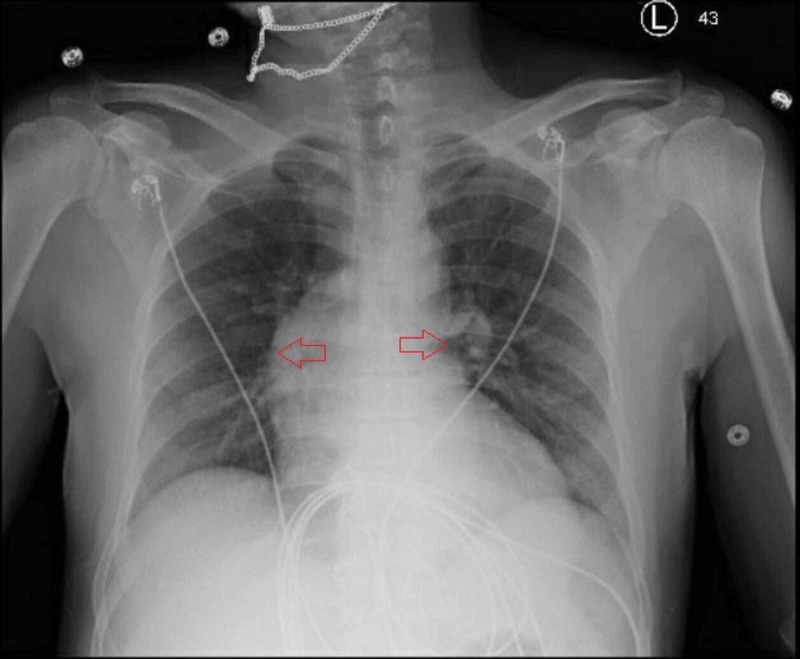
Chest X-ray showing clear lungs and a prominent mediastinum (red arrows).

**Figure 2:**
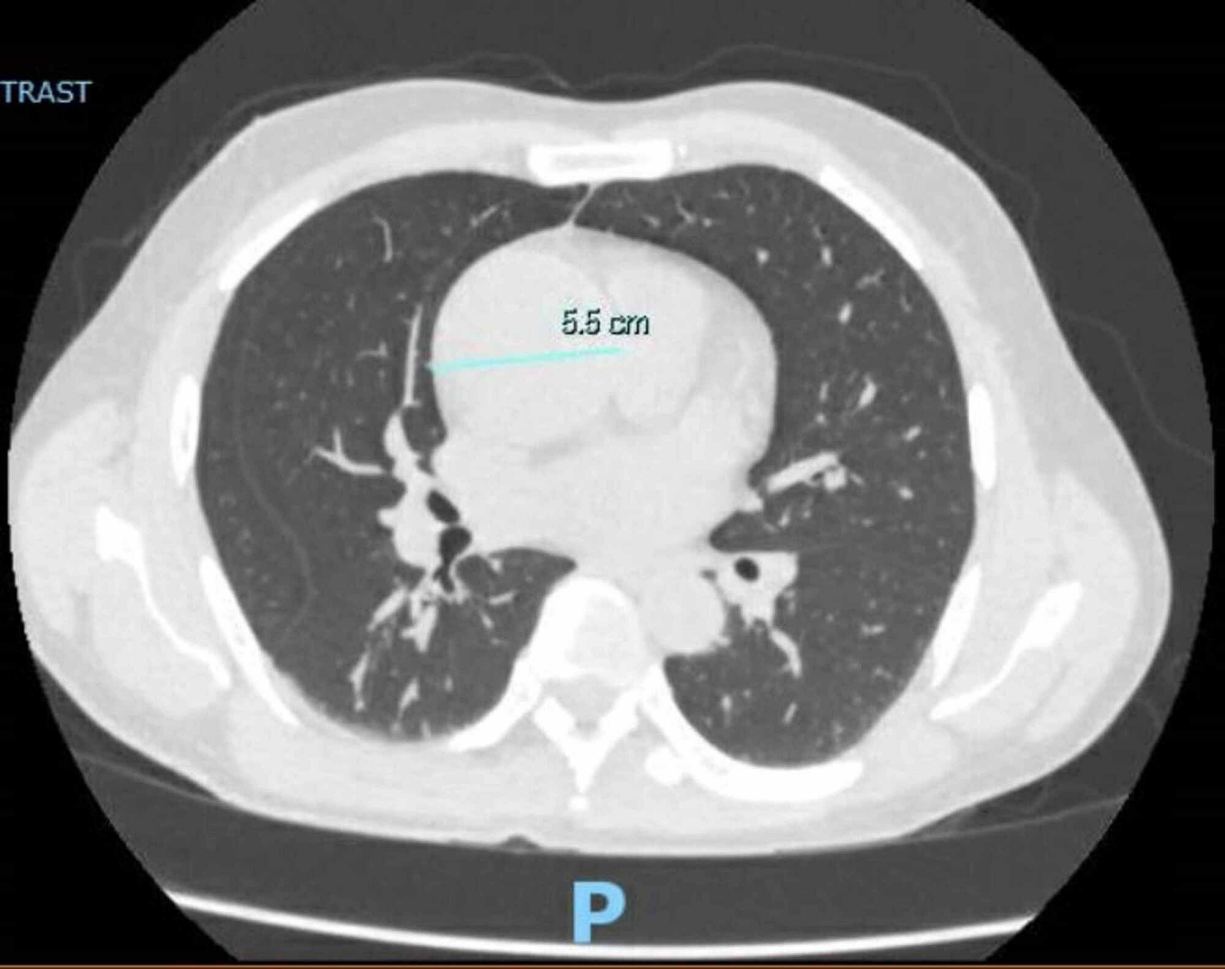
Computerized tomography (CT) angiogram of the chest showing ascending aortic aneurysm up to 5.5 x 5.2 cm in diameter without aortic dissection or intramural hematoma.

**Figure 3:**
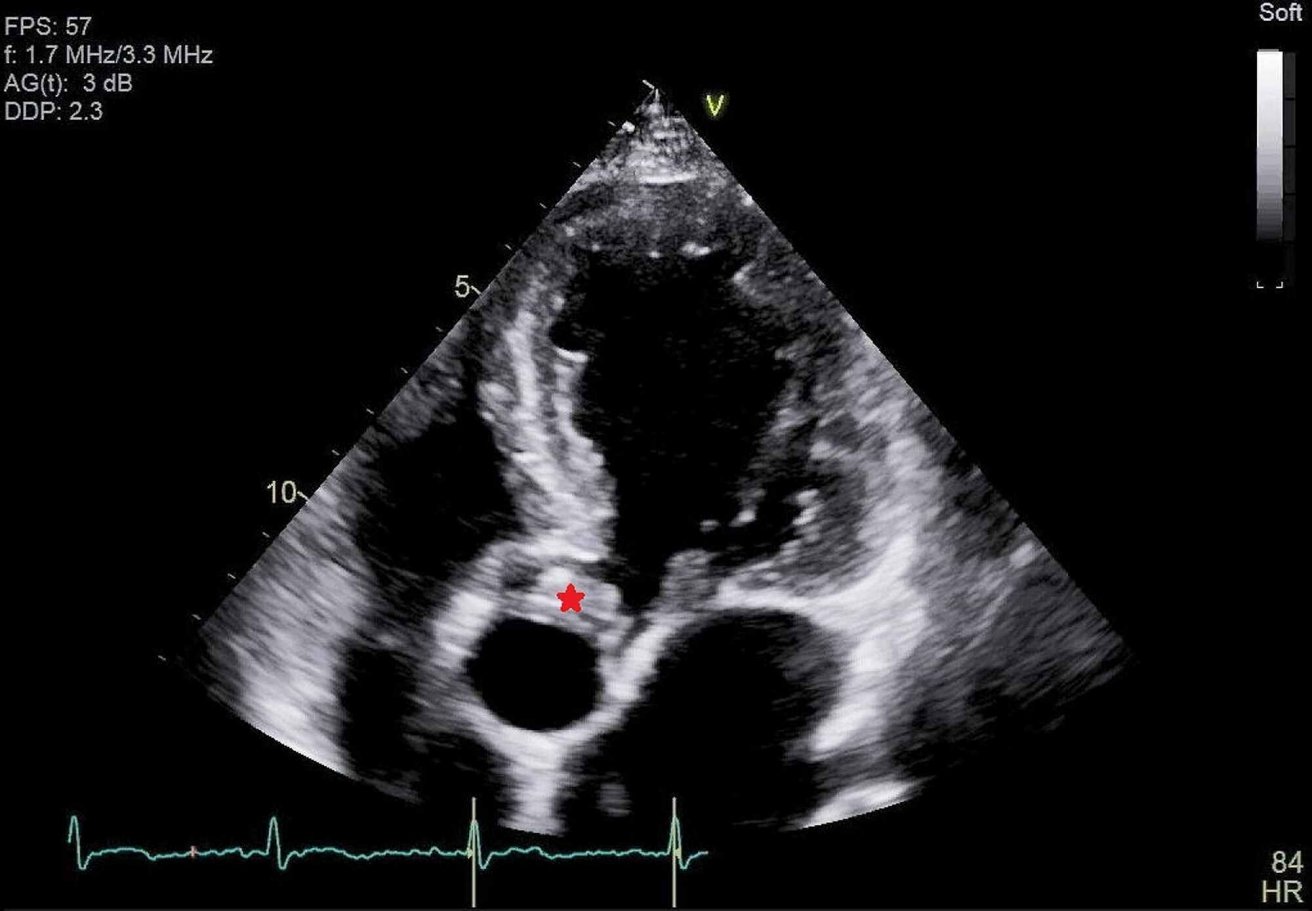
Transthoracic echocardiogram (apical five-chamber view) on admission showing a large aortic valve vegetation (red asterisk).

At the emergency room, he was febrile with a temperature of 100.5°F (38°C), normal blood pressure, and oxygen saturation at 99% on ambient air. On physical examination, he appeared to be developed and well-nourished. He had some dental caries but no obvious abscess. His lung sounds were clear to auscultation. He was found to have a harsh blowing murmur best heard at the right upper sternal border. Laboratory tests revealed mild leukocytosis with left shift (white blood cell count of 12.94 K/uL and neutrophil 83.3%). He did not have abnormal blood cells on peripheral smear to suggest a hematologic disorder. He had normal kidney and liver function tests. Elevation of inflammatory markers was noticed, including erythrocyte sedimentation rate of 33 mm/hr and serum C-reactive protein of 5.85 mg/L. Human immunodeficiency virus and syphilis screening tests were negative. The CXR showed clear lungs and a prominent mediastinum (Figure 1). He underwent computerized tomography (CT) angiogram of the chest, abdomen, and pelvis, which showed ascending aortic aneurysm up to 5.5 x 5.2 cm in diameter without aortic dissection or intramural hematoma (Figure 2). Transthoracic echocardiogram demonstrated aortic valve vegetations (Figure 3) and severe aortic insufficiency.

Aerobic and anaerobic blood culture bottles obtained in the emergency room grew after 16 hours of incubation, with Gram-positive cocci in pairs noticed on microscopy. *Streptococcus* species was detected through the polymerase chain reaction technique using BioFire® FilmArray® Blood Culture Identification Panels (BioFire Diagnostics, Salt Lake City, Utah, USA). The organism was further identified by matrix-assisted laser desorption ionization-time of flight mass spectrometry (MALDI-TOF MS) (bioMérieux, Marcy l'Etoile, France) as *S. anginosus*. Using ETEST® antimicrobial susceptibility test strips, the isolate was susceptible to penicillin (minimum inhibitory concentration [MIC] = 0.047 μg/mL) and ceftriaxone (MIC = 0.25 μg/mL). Kirby-Bauer disc-diffusion antibiotic susceptibility testing revealed that it was also susceptible to clindamycin, erythromycin, vancomycin, and levofloxacin. Therefore, it was confirmed that he had aortic valve IE secondary to *S. anginosus* bacteremia. Yet, the dental consultation did not find an oral source of IE. The patient was started on IV ceftriaxone 2 grams every 24 hours and IV gentamicin 220 mg every 24 hours.

**Figure 4:**
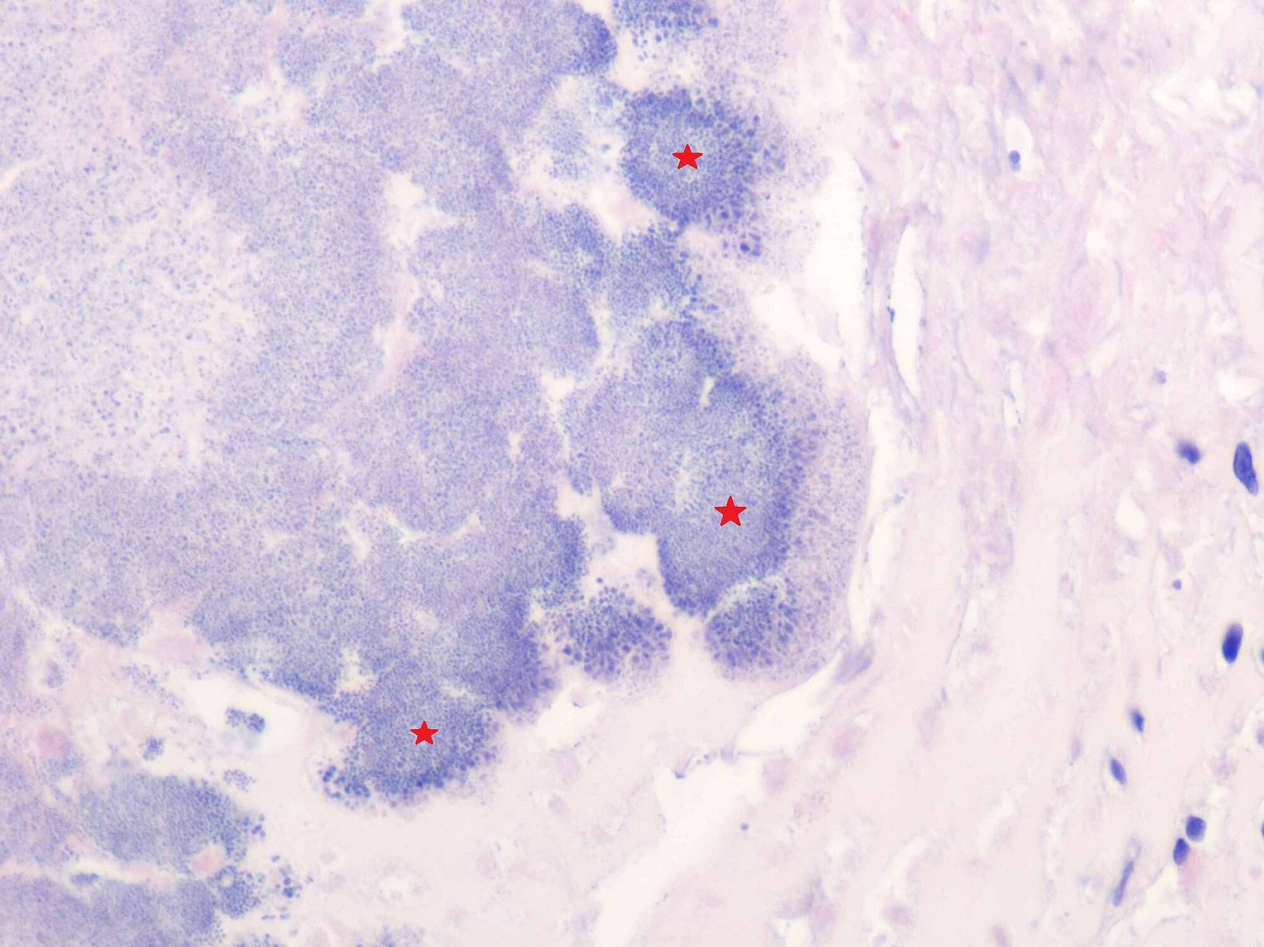
Bacterial colonies on the aortic valve. High-power microscopic view (hematoxylin and eosin stain, original magnification x600) demonstrated bacterial colonies (red asterisks) on the aortic valve.

Intra-operative transesophageal echocardiogram showed that the patient had a bicuspid aortic valve. He underwent surgery for excision of infected bicuspid aortic valve and aortic valve replacement utilizing a 23-mm bovine pericardial Carpentier-Edwards bioprosthesis. The aortic valve leaflets consisted of two partially calcified, tan-yellow, valvular tissue measuring 3.0 x 3.0 x 1.2 cm in aggregate. High-power microscopic view demonstrated bacterial colonies on the aortic valve (Figure 4). No obvious aortic root abscess could be seen. The cardiothoracic surgeon resected the ascending aortic aneurysm and replaced with a 28-mm Hemashield interposition vascular graft.

The surgical culture from the aortic vegetations also grew *S. anginosus*, which was susceptible to penicillin and ceftriaxone. He underwent upper endoscopy and colonoscopy, which failed to show any gastrointestinal (GI) malignancy. His recovery from surgery was optimal. He completed the antibiotics course of IV ceftriaxone and gentamicin for two weeks after the surgery in the hospital. Even though the pathology report did not show bacterial involvement of the aortic aneurysm, the infectious diseases team made the decision to extend the patient’s ceftriaxone regimen to six weeks to avoid any risk of developing untreated mycotic aneurysm. He was subsequently discharged to a rehabilitation facility on another four weeks of IV ceftriaxone. He was followed closely in our Infectious Disease Clinic. Blood culture showed no growth of bacterium two months after discharge.

## Discussion

SAG, including *S. anginosus*, *S. constellatus*, and *S. intermedius*, are gram-positive, catalase-negative cocci and nonmotile facultative anaerobes that have typically small colonies (≤0.5 mm of diameter) [1]. They can exhibit different hemolysis patterns on sheep blood agar and displace different Lancefield antigens; therefore, identification of the group could be challenging [3]. Those three species can be differentiated by phenotypic methods that correlate well with molecular taxonomic techniques developed by Whiley et al. [3]. However, non-sequence-based methods for identification, such as matrix-assisted laser desorption ionization-time of flight (MALDI-TOF) mass spectrometry, is a fast and reliable way to detect any given organism [4]. Nucleic acid amplification assay such as 16S rRNA gene sequencing is another way to further identify the species. Recent studies based on multi-locus sequence analysis (MLSA) showed that *S. anginosus* can further classify as *S. anginosus subsp. anginosus*, *S. anginosus subsp. whileyi*, *S. anginosus* *genomosubsp*. *AJ1*, and a potentially new strain called *S. anginosus* *genomosubsp. vellorensis* [5,6]. On the other hand, *S. constellatus* has three subspecies, which are *S. constellatus subsp*. *constellatus*, *S. constellatus subsp*. *pharyngis*, and *S. constellatus* *subsp. viborgensis* [5]. SAG is ubiquitously presented in human skin, oral, GI, and genitourinary microbiota and is considered to be harmless commensal [3]. When they become pathogenic, *S. intermedius* can form abscesses of the brain and liver, whereas both *S. anginosus* and *S. constellatus* can be isolated from a wider range of sites (such as head and neck, GI, genitourinary, and respiratory tracts) and infections [3]. Anginosus group, along with mitis, mutans, salivarius, and sanguinis groups, constitutes the most clinically significant species of viridans streptococci [7]. However, unlike other viridians streptococci, SAG is an unusual agent of IE [8]. In a study of 377 cases of IE, there were only six patients who had SAG infection and all of them attributed to *S. anginosus* [9].

The HANDOC score is a sensitive tool to determine if an echocardiogram is indicated in patients with non-β-hemolytic streptococci bacteremia [10]. There were 105 SAG bacteremia cases in their cohort, but none of them have endocarditis. Hence, if the etiology of bacteremia is from SAG, the HANDOC point will have to subtract 1 point, thus implying the rarity of SAG IE. Most patients with IE due to SAG have underlying medical diseases. In the current literature, there are case reports of SAG IEs associated with hepatic abscess, brain abscess, splenectomy state, and colon cancer [11-13]. In this case, the only risk factor we identified was the presence of bicuspid aortic valve despite the patient not having any proceeding dental procedure. The chief complaint of the patient was persistent dry cough. Yet, neither the CXR nor the chest CT showed pneumonia. The large size of the aortic aneurysm could possibly compress the trachea and main bronchus and induce cough [14]. SAG IE frequently involves the left side of the heart valves (59% in mitral valve and 45% in aortic valve) [15]. Valvular insufficiency is observed in 90% of the cases of SAG IE [15]. If the infection damages the vessel wall causing dilatation of the arterial wall, it can form a mycotic aneurysm that is known to be a complication of IE. A mycotic aneurysm was not observed in our patient; he suffered from aortic regurgitation that further results in the dilation.

Beta-lactams are the treatment of choice for SAG infections. Almost all isolates of SAG are susceptible to penicillin; however, intermediate susceptibility was seen in 15% of isolates [16]. In a Japanese study, the non-susceptible rates to erythromycin, clindamycin, and azithromycin were about 8%, 5%, and 4%, respectively [17]. According to the American Heart Association (AHA) guidelines, the treatment regimen for adult native valve endocarditis from penicillin-susceptible viridans streptococci (defined by MIC ≤ 0.12 mcg/mL) includes four weeks of aqueous penicillin G, ampicillin, or ceftriaxone IV [18]. The two-week regimen of aqueous penicillin G or ceftriaxone IV plus gentamicin IV has a synergic effect and is also considered an acceptable alternative [19]. Some of the indications for early valve surgery in left-sided native valve endocarditis according to the AHA recommendation include valve dysfunction resulting in heart failure, IE caused by fungi or highly resistant organisms, IE complicated by heart block or aortic abscess, persistent bacteremia, recurrent emboli, and persistent or enlarging vegetations despite appropriate antibiotic therapy, severe valve regurgitation, and mobile vegetations >10 mm [18]. Even though having a bicuspid valve carries a higher risk for endocarditis, there is insufficient evidence and literature on the efficacy of antibiotic prophylaxis given prior to any dental manipulation that involves the mucosa or gingival tissues [20]. SAG infections generally have favorable outcomes. However, the progression to bacteremia can carry a poor prognostic factor [16]. The mortality rate for SAG endocarditis is 14% according to a French study [15].

## Conclusions

SAG has a propensity in forming abscesses and invasive pyogenic infections, but it is a rare cause of endocarditis. Our case report showed that SAG endocarditis can occur on a bicuspid aortic valve in an immunocompetent host. Most species of SAG are penicillin-sensitive, but the treatment outcomes can be compromised by bacteremia or endocarditis.
